# Occurrence of *Trichophyton verrucosum* in cattle in the Ningxia Hui autonomous region, China

**DOI:** 10.1186/s12917-020-02403-6

**Published:** 2020-06-10

**Authors:** Yanan Guo, Song Ge, Haifeng Luo, Atif Rehman, Yong Li, Shenghu He

**Affiliations:** 1 Department of Veterinary Clinical Sciences, School of Agriculture, Ningxia University, Yinchuan, 750021 Ningxia China; 2 Department of Life Science and Technology, Ningxia Polytechnic College, Yinchuan, 750021 Ningxia China

**Keywords:** Fungi, Isolation, Identification, Ningxia Hui autonomous region, China

## Abstract

**Background:**

Ningxia Hui Autonomous Region is an important cattle breeding area in China, and cattle breeding bases are located in this area. In Ningxia, dermatophytes have not been paid attention to, so dermatophytosis is becoming more and more serious. For effective control measures, it is important to determine the disease prevalence and isolate and identify the pathogenic microorganism.

**Results:**

The study showed the prevalence of dermatophytes was 15.35% (74/482). The prevalence in calf was higher than adult cattle (*p* < 0.05). The morbidity was the highest in winter compared with autumn (*p* < 0.0001), summer (*p* < 0.05) and spring (*p* < 0.0001). The prevalence in Guyuan was the highest compared with Yinchuan (*p* < 0.05) and Shizuishan (*p* < 0.05). The incidence of lesions on the face, head, neck, trunk and whole body was 20.43, 38.71, 20.43, 10.75 and 9.68%, respectively. From all samples, the isolation rate of *Trichophyton* was highest (61.1%). The phylogenetic tree constructed showed that the 11 pathogenic fungi were on the same branch as *Trichophyton verrucosum*.

**Conclusions:**

This study reports, for the first time, the presence of *Trichophyton verrucosum* in cattle in Ningxia and showed that the incidence of dermatophytosis is related to different regions, ages and seasons. A better knowledge of the prevalence of dermatophytosis of cattle may allow the adoption of more efficient control measures and prophylaxis.

## Background

Dermatophytosis in animals are mostly caused by Dermatophytes that is a group of closely related organisms which can invade the stratum corneum of the epidermis [1, 2]. Dermatophyte infections are a major health problem both in humans and animals around the world, which can be transmitted from animal to animal and from animals to humans, causing outbreaks among exposed individuals [3, 4].

Approximately more than 40 kinds of dermatophytes have been discovered, and they mainly infect skin, hair, and other tissues, causing dander increased, hair removal, exudation, folliculitis, itching and other clinical signs [5, 6]. They are the causative agents of ringworm, also commonly referred to as dermatophytosis or *Tinea* [7]. Dermatophytes are classified as anthropophilic, zoophilic, and geophilic based on their primary reservoirs [8–10]. Anthropophilic are primarily associated with humans and rarely infect animals [8, 10]. Zoophilic dermatophytes usually infect animals or are associated with animals but occasionally infect humans [8, 11, 12]. Geophilic occasionally can be pathogenic for humans or animals [8, 11, 12].

Dermatophytosis of cattle are one of the pathogens affecting public health. In particular, worker infections caused by *Trichophyton rubrum* are often common because these people are in direct contact with infected cattle [7]. The infection caused by *Trichophyton verrucosum* (*T. verrucosum*), *Trichophyton rubrum, Trichophyton mentagrophytes*, *Trichophyton simii* and *Microsporum gypseum* has been reported [7, 13].

Calves belonging to either sex, breed, age in all kinds of farm condition are considered highly exposed to *T. verrucosum* infection [7, 14]. Calves are more affected than adults because they have not specific immunity yet from natural exposure to these fungi [3]. Transmission occurs through direct contact with sick animals, and indirect transmit, such as from an infected environment, poor nutrition and livestock mismanagement, as well as lack of sanitation routines, which leads to facilitate spreading of fungi [3]. The infection is common in those countries which have a hot and humid climate. In temperate areas, the peak of infection usually occurs in Summer and Winter [3, 15]. In China, cases of *T. verrucosum* infection in cattle and humans are mainly reported in Xinjiang Uygur Autonomous Region in Western China [16].

The Ningxia Hui Autonomous Region is an important cattle breeding area in China. There are five cities (Shizuishan, Yinchuan, Zhongwei, Wuzhong, Guyuan) in Ningxia, and four of them (Yinchuan, Wuzhong, Shizuishan and Guyuan) are the main cattle breeding bases. Only a few cases of *T. verrucosum* infection in cattle have been reported in Ningxia Hui Autonomous Region in Western China.

Although the dermatophytosis is rarely fatal, it has a negative impact on animal growth and meat production and cause the poor quality of rawhide materials in view of hides and skins being affected and destroyed by dermatophytes, which can cause huge economic losses to animal husbandry [3, 17]. So, this study is a great step towards the prevention and control of cattle fungal skin diseases in Ningxia Hui Autonomous Region.

## Results

### Epidemiological investigation results

**Fig. 1:**
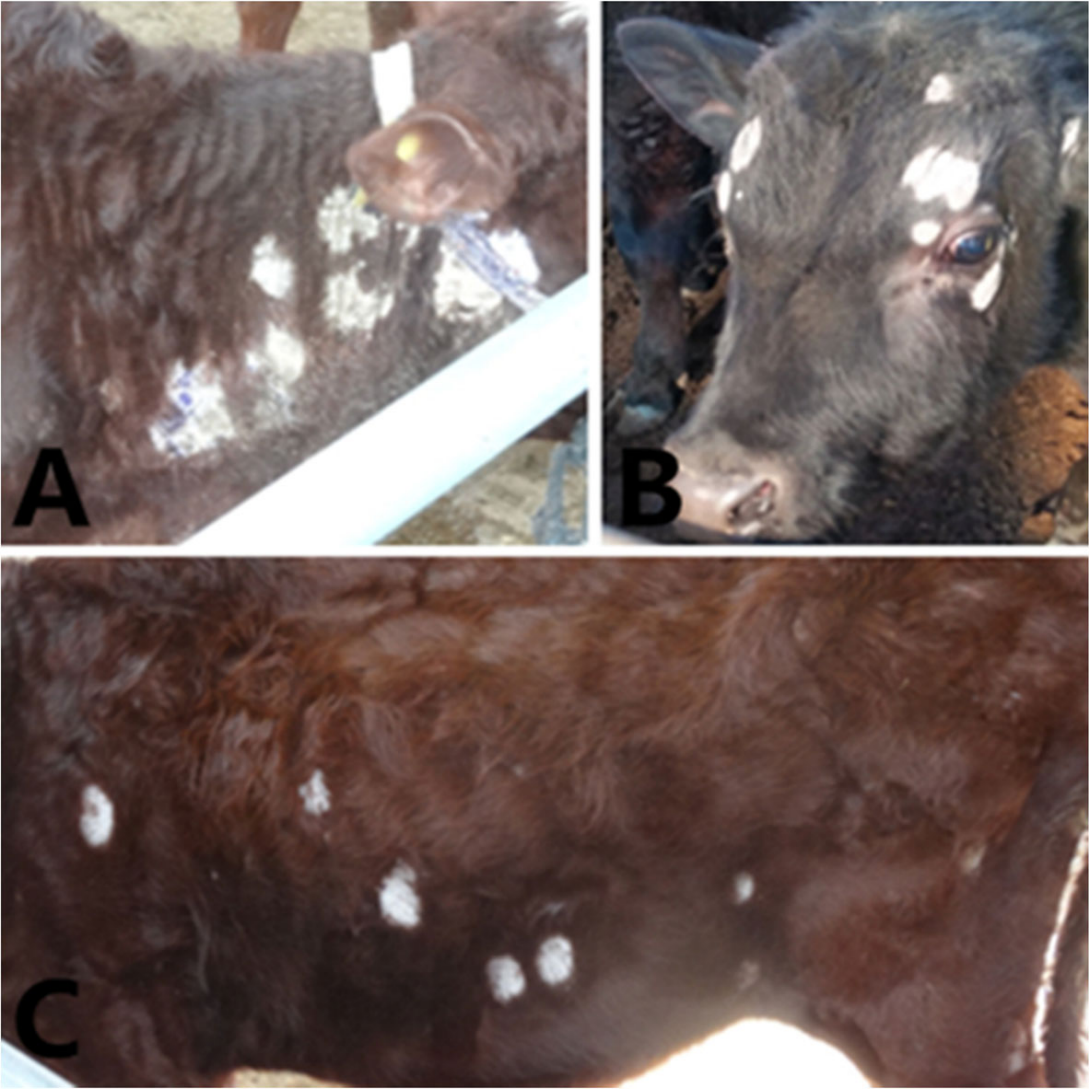
Dermatophytosis in cattle. **a** Grayish-white desquamations around the neck. **b** Grayish-white desquamations around the head. **c** Grayish-white desquamations around the trunk

A total of 482 beef cattle from 4 different cities of Ningxia province were investigated, and the results of the study were showed in Table 1. The clinical signs of cattle with dermatological diseases were similar. The lesions were mainly found on the neck (Fig. 1a), head, face (Fig. 1b) and trunk (Fig. 1c).

**Table 1:** Prevalence of disease in different cities of Ningxia

Cities	No. of skin lesions suggestive of dermatophyte	No. of Infected samples	Total samples	Prevalence (%)	*p* value
Yinchuan	9	9	88	10.23	*p* = 0.0104 < 0.05(Chi-square 6.5723) for Guyuan vs Shizuishan, *p* = 0.0208 < 0.05(Chi-square 5.3429) for Guyuan vs Yinchuan
Shizuishan	17	17	154	11.04	
Wuzhong	21	21	120	17.50	
Guyuan	27	27	120	22.50

The prevalence of disease in Yinchuan was 10.23% (9/88), Shizuishan was 11.04% (17/154), Wuzhong was 17.50% (21/120) and Guyuan was 22.50% (27/120), respectively. The prevalence of disease in Guyuan was the highest, followed by Wuzhong, Shizuishan and Yinchuan. Meanwhile, Guyuan was significantly different(*p* < 0.05), as compared to Yinchuan and Shizuishan, but the prevalence of disease in Wu Zhong was lower than Guyuan nonsignificantly (*p* > 0.05). In addition, Yinchuan and Shizuishan were also similarly nonsignificant (*p* > 0.05). (Table 1).

Among the investigated herds (*n* = 482), the prevalence of disease in calf were 10.82% (53/288) and the prevalence of disease in adult cattle were 18.40% (21/194). The prevalence of disease in calf were higher than the adult cattle and significantly different (*p* < 0.05) (Table 2).

**Table 2:** Prevalence of disease in different ages and seasons (*n* = 482)

Variables	No. of Infected samples	Total samples	Prevalence%	*p* value
Age				
Calf	53	288	18.40	*p* = 0.0236 < 0.05(Chi-square 3.9726)
Adult cattle	21	194	10.82	
Season				
Winter	36	482	7.47	*p* < 0.0001(Chi-square16.9933) for winter vs autumn, *p* = 0.0182 < 0.05 (Chi-square 5.5725) for winter vs summer, *p* < 0.0001 (Chi-square 15.4320) for winter vs spring
Spring	10	482	2.07	
Summer	19	482	3.94	
Autumn	9	482	1.87	

The highest infection rate was found in winter compared with autumn (*p* < 0.0001), summer (*p* < 0.05) and spring (*p* < 0.0001). The prevalence of infection was higher in summer than spring and autumn, but they were not significantly different (*p* > 0.05) by statistical analysis (Table 2).

**Fig. 2:**
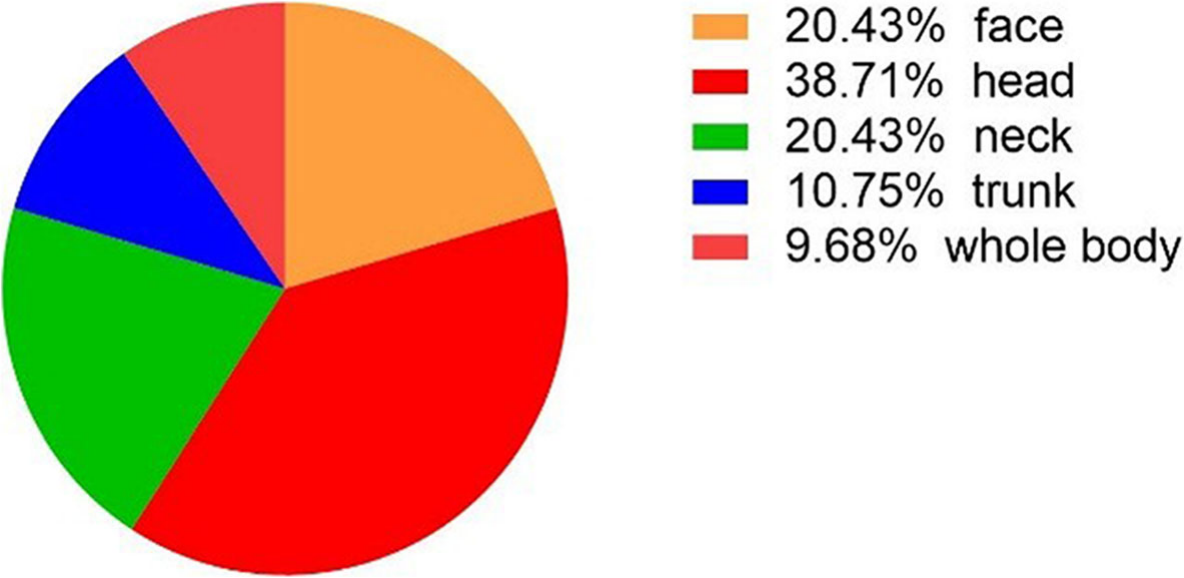
The distribution ratio of skin signs in different body sites

The ringworm lesion appeared on the skin, and the sign were scattered in different body sites. The prevalence of lesions on the face, head, neck, trunk and whole body was 20.43, 38.71, 20.43, 10.75 and 9.68%, respectively. (Fig. 2).

### Microscopic examination results

The 74 samples that were coming from skin desquamation and hair of skin lesions of beef cattle were collected from Yinchuan (*n* = 9), Shizuishan (*n* = 17), Wuzhong (*n* = 21), Guyuan (*n* = 27) cities in Ningxia province in China., then observed with microscope. All samples were observed septate hyphae in skin scales and ectothrix spores in hair, parasites and parasite eggs were not found. Percentage of positivity of fungi by direct microscopic examination was 100% (74/74).

### Isolation and culture results

**Fig. 3:**
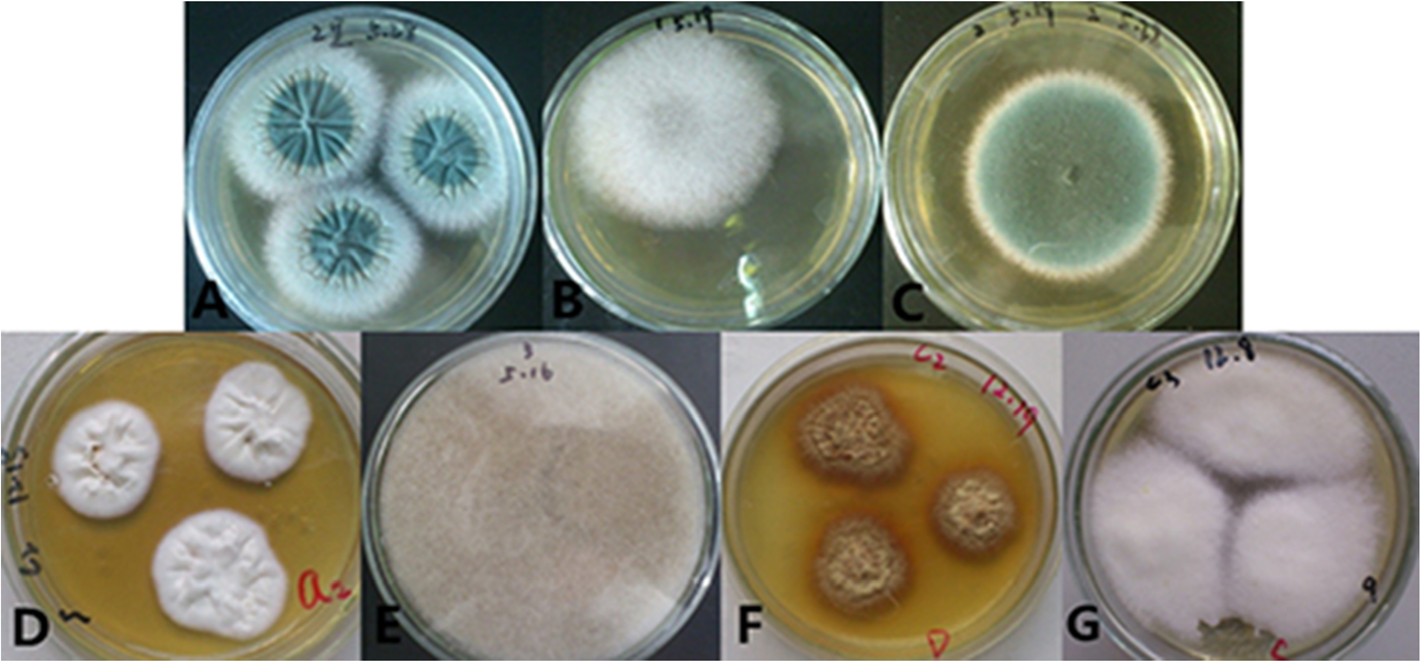
Colony morphology of fungi isolated and cultured on SDA medium. **a***Alternaria*. **b***Fusarium.***c***Aspergillus*. **d***Trichophyton*. **e***Mucor*. **f***Epicoccum*. **g***Lichtheimia*

The isolated fungi were initially classified according to the colony morphology on SDA medium. There are seven species of fungi cultured with 18 samples, including 4 strains of *Alternaria* (Fig. 3a), 4 strains of *Fusarium* (Fig. 3b), 7 strains of *Aspergillus* (Fig. 3c), 11 strains of *Trichophyton* (Fig. 3d), 4 strains of *Mucor* (Fig. 3e), 4 strains of *Epicoccum* (Fig. 3f), 7 strains of *Lichtheimia* (Fig. 3g).

### Identification results of *Trichophyton verrucosum*

Eleven strains of *Trichophyton* had similar morphological structures, and their colonies growth was slow. After 1 week of culture, white fluffy colonies were formed and were firmed in texture. Microscopy showed that mycelium was filiform, 2 - 6 μm pear-shaped and small conidia grow along with the mycelium to form antler-like hypha. After 2 weeks, the colonies formed some white powdery colonies with no pigment produced, showing typical chain chlamydospores, curved mycelium with a diameter of 3 - 4 μm were observed. According to the results of culture, isolation and morphological observation, they were initially identified as *T. verrucosum*. The 11 *Trichophyton* strains isolated were named NXGY1, NXGY2, NXYC1, NXYC2, NXYC3, NXWZ1, NXWZ2, NXWZ3, NXSZS1, NXSZS2, NXSZS3.

### *Trichophyton* gene identification

**Fig. 4:**
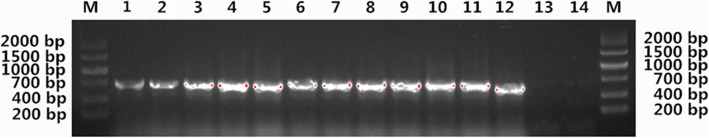
Agarose electrophoresis of amplified ITS from *Trichophyton.* (M: DL2000 Maker; 1–11: The amplified ITS fragments of NXGY1、NXGY2、NXYC1、NXYC2、NXYC3、NXWZ1、NXWZ2、NXWZ3、NXSZS1、NXSZS2、NXSZS3. 12:Positive control. 13: Negative control. 14: Blank control)

The intergenic spacers of 11 *Trichophyton* strains and *T. verrucosum* ATCC 42898 were amplified by PCR amplification. The amplified products were analyzed by agarose gel electrophoresis. The results showed that the gene amplification showed about 650 bp DNA fragments (Fig. 4).

### Genetic analysis of *Trichophyton*

**Fig. 5:**
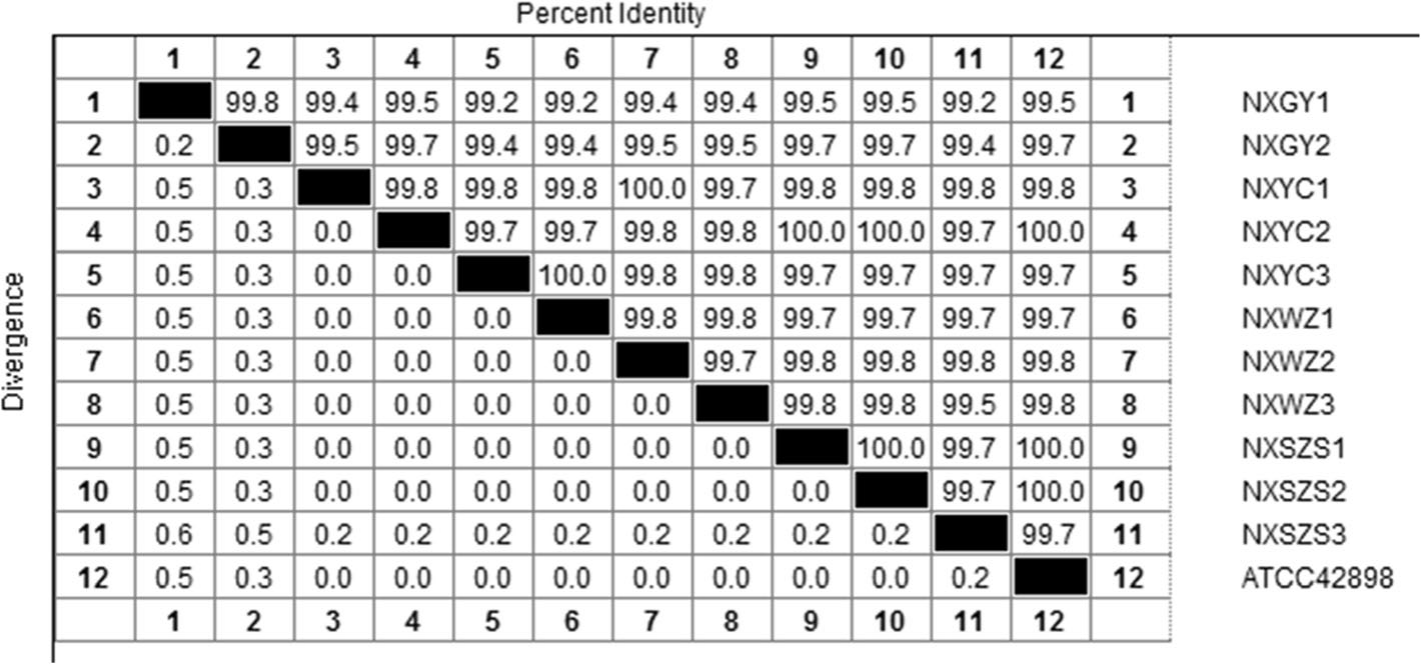
Amplification sequence similarity test results of 11 strains of *Trichophyton*

**Fig. 6:**
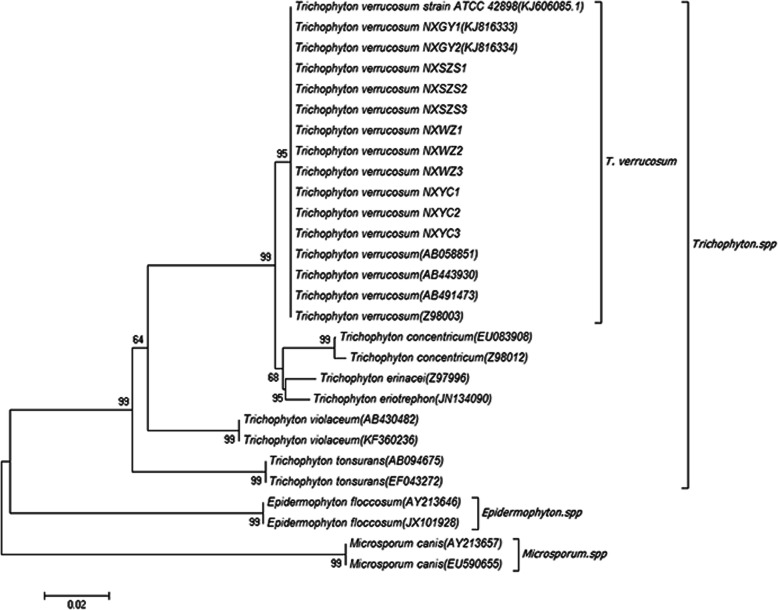
Phylogenetic tree constructed with the program Neighbor—Joining (NJ) based on the amplicons of ITS1 and ITS4 region

The amplification sequence of 11 *Trichophyton* strains and *T. verrucosum* ATCC 42898 were analyzed by the Sequence Distances method of MegAlign software. The similarity was above 99.2% (Fig. 5). The phylogenetic tree was constructed based on the gene sequences of ITS1 and ITS4 of 11 *Trichophyton* strains, and all sequences were compared with *Trichophyton verrucosum*, *Trichophyton concentricum*, *Trichophyton violaceum*, *Epidermophyton floccosum* and *Microsporum canis* strains. The results were shown in Fig. 6. The phylogenetic tree constructed showed that the 11 pathogenic fungi were on the same branch as *T. verrucosum*. The NCBI GenBank accession numbers obtained by NXGY1 and NXGY2 are: KJ816333 and KJ816334 (Fig. 6).

## Discussion

Clinical signs and survey results showed that lesions were mainly found on the head and neck, especially around the eyes and face. For more serious beef cattle, lesions could appear in the whole body, which is consistent with previous reports [13, 18]. The reason for head and neck that is the main site of disease is not yet clear and may be related to the organization and husbandry of beef cattle. Firstly, skin tissue around the eyes of beef cattle is soft, which is more susceptible to fungal attachment and growth. Secondly, the beef cattle in Ningxia are mainly intensive farming patterns, so the beef cattle suffer frictional damage to the head and neck by the railings during feeding, then the fungal could infect and grow on the damaged skin, which is absent of the epidermis defense.

Calves are more susceptible than adult cattle, that is consistent with previous reports [13, 19]. The higher prevalence of calves is related to its undergrowth skin and hair, sebaceous gland less secretions, incomplete skin micro-ecological flora and the immune system hypoplasia [13]. In our study, we got the same results.

In order to identify the influence of the humid environment, the traceable climate data from the Ningxia Meteorological Service Network (http://www.qx121.com.cn/) during the study period was analyzed, and the consistent correlation between morbidity and precipitation was noticed. The prevalence rate is higher in the cities (Guyuan and Wuzhong), which has the high precipitation. Yinchuan and Shizuishan are moderate temperate arid areas with annual precipitation of 180–200 mm. Wuzhong is a moderate temperate semi-arid areas with annual precipitation of 280–300 mm. Guyuan is moderate temperate semi-humid areas with annual precipitation above 400 mm..Regional analysis found that the prevalence of beef cattle skin disease was higher in semi-humid areas (Guyuan) than in semi-arid areas (Wuzhong). Semi-arid areas are higher than arid areas (Yinchuan and Shizuishan). These results show that beef cattle are more susceptible to skin dermatophytes and more conducive to the spread of pathogens in areas with more precipitation. This result is consistent with the incidence of dermatophytosis in high humidity areas compared to low humidity areas [11].

In our study, the prevalence rate is high in summer and winter, and previous studies reported the similar results [15]. Summer is hot, with strong sunshine and humidity, which accelerates the metabolism of the skin cells increasing a lot of sebum secretion, the large amount of sweat and other wastes discharged from the skin. So, the surface environment of the skin is changed, which weakens the disease resistance of the beef cattle and promotes the growth of the fungus. Winter is cold, the herd gather together, which promote the spread and outbreak of dermatophytosis. Meanwhile, the prevalence is higher in winter than summer. The reason may be that cattle gather together in winter, and they collide with each other to cause the spread of fungal diseases more easily.

Out of the 482 cattle examined, 74 had skin lesions suggestive of dermatophytosis. Samples from these 74 animals processed for fungi detection by direct microscopic examination and all were positive for fungi. Isolation and identification of 18 samples which were coming from severely affected cattle showed that not only pathogenic fungi *Trichophyton verrucosum* were isolated, but also saprophytic fungi of *Lichtheimia*, *Aspergillus* and *Mucor* were isolated. These saprophytic fungi could also cause pathogenicity and strengthen the damage to skin tissue [2]. *Fusarium* is widely found in soil and can produce toxins to contaminate animal feed, which can cause foot rot in cattle after feeding [5]. *Alternaria* is mainly parasitic on plants and can cause economic plant diseases [2]. *Epicoccum* is a kind of plant fungi. It can produce conidia and antifungal compounds on the surface of senile or newly dead plant tissues [2, 6]. Among all fungi, the *Trichophyton verrucosum* were isolated mostly, so the main pathogenic fungi causing cattle dermatosis are *Trichophyton verrucosum.* These rotten fungal (*Lichtheimia*, *Aspergillus* and *Mucor*) infections were induced after *Trichophyton verrucosum* infection, which aggravates the condition of the diseased cattle. *Lichtheimia*, *Aspergillus* and *Mucor* appear in the collected samples, which may be due to the presence of these fungi in the litter or feed of the cattle. When sleeping or eating feed, the lesions are itchy. Therefore, when it is rubbed with forage, it causes adhesion and infection of these fungi in the lesion. Out of 11 strains of *Trichophyton verrucosum* isolated from 18 samples, the isolation rate of *Trichophyton verrucosum* was 61.1%, which is in accordance with Courtellemont L [20] report.

In recent years, with the rapid development of intensified farming techniques in Ningxia and the widespread use of glucocorticoids and antibiotics on animals, the immunity of animals is often greatly reduced, so that the incidence of some saprophytic fungi diseases is increasing year by year [21]. As *Trichophyton verrucosum* can survive in the environment for 5–7 years [22, 23], soil and contaminated practices, even homes, can be sources of infection for animals, which should be concerned.

Microscopic examination and culture in dermatophyte detection were the lower sensibility. These two detection methods can only determine the presence of fungi in the collected samples and cannot determine the species of the fungus. At present, PCR technology combined with DNA sequence analysis technology is commonly detection method used to identify fungal species. The internal transcribed spacer (ITS) is relatively conserved in long-term evolution and has large evolutionary differences among different species, so the internal transcribed spacer is often used for species classification studies [24–26]. The similarity analysis and homology analysis of the ITS sequences of 11 *Trichophyton* strains showed that the 11 strains of *Trichophyton* were *Trichophyton verrucosum*. It is indicated that *Trichophyton verrucosum* is widely present in Ningxia.

Currently, Norway and Sweden have reported corresponding sputum vaccines to control this disease [27, 28]. However, in China, there is no approved vaccine to prevent the disease. Without the vaccine or prevention method the current occurrence of dermatosis is more and more seriously in China, especially in the Ningxia Hui Autonomous Region, which has a large cattle production. Therefore, we need to improve the feeding environment of the cattle farm, isolate the infected cattle, give sufficient sunshine, regular medicated bath, keep the cattle farm dry and give appropriate therapeutic drugs to alleviate the disease, in order to prevent infection caused by *Trichophyton verrucosum*. The farm managers and veterinarians should be educated for the prevention in summer and winter. In summer, it is necessary to drain well, keep the cattle farm dry and ensure the sanitation and cleanliness of the shed, isolate sick cattle and disinfect them regularly. In winter, it is necessary to keep shed warm, air flow, isolate sick cattle and give sufficient sunshine time.

Cattle ringworm causes high economic losses. Fungal skin diseases not only affect the growth and development of cattle but also cause skin damage, then eventually cause high economic losses, so more in-depth study is needed in future.

## Conclusions

This study reports, for the first time, the presence of *T. verrucosum* in cattle in Ningxia Hui Autonomous Region and showed that the incidence rate of cattle fungal skin disease is higher in summer and winter, and calf are easy to be infected than adult cattle, and the different strains of *T. verrucosum* are in the same position in the Phylogenetic tree, that showed all the strains come from same source.

## Methods

### Cattle and sample preparation

The total number of beef cattle investigated was 482, including 288 cattle ≤6 months, 194 cattle > 6 months. This study investigated beef cattle farms in Yinchuan (*n* = 88), Wuzhong (*n* = 120), Shizuishan (*n* = 154) and Guyuan (*n* = 120) in Ningxia.

Cattle were observed whether they had dermatophytosis, and diagnosed with lesions such as alopecia, scaling, crusts, bleeding, hair dullness, emaciation and itching. The number of groups, the number of cattle affected, age, lesion site and the time of onset were recorded.

### Sample collection

Skin desquamation and hair collection were performed at the junction between healthy and affected skin lesions, after cleaning with ethyl alcohol 70% using a sterile scalpel blade [29]. The samples were preserved in sterile sealed bags and sent for fungal examination laboratory in School of Agriculture, Ningxia University.

### Microscopic examination

Preserved samples in sterile sealed bags were transferred to slides and added 1 drop of 10% KOH solution to soften the dander for 5 min and then observed under an optical microscope [30].

### Mycological culture

Eighteen samples from cattle with neck, head, face and trunk lesions were inoculated onto the Sabouraud Dextrose Agar (SDA) culture medium (Qingdao haibo, Qingdao, China) with 3 points of inoculation [31, 32]. The samples were cultured in the incubators at 30 °C and 37 °C for 1–2 weeks. After that, the colony growth was observed every day. When the new hyphae were grown, they were inoculated in SDA medium, and cultured in a constant temperature incubator at 30 °C and 37 °C [31, 32]. Preliminary species classification of fungi was made, according to the growth rate, colony form, color change of culture medium, mycelium and conidial morphology.

### Gene identification

Approximately 100 mg of fresh mycelium was pulverized in liquid nitrogen to cause initial decomposition. The fungi’s genome DNA were extracted with a DNA extraction kit (Takara, Japan). The ITS1 (5′-TCCGTAGGTG AACCTGCGG-3 ‘) and ITS4 (5’-TCCTCCGCTTATT GATATGC-3′) specific primers of pathogenic fungi were used for internal transcribed spacer [4, 30, 32]. The PCR reaction mixture was 25 μl (2.5 μl,10 × PCR Buffer, 2.0 μl dNTP Mixture, 0.125 μl rTaq, 1 μl primer, 0.5 μl DNA, plus ddH2O to 25 μl) (TaKaRa, Japan) [4, 30, 32]. The reaction mixture was incubated at 94 °C for 5 min, then subjected to 30 cycles of 94 °C for 30 s, 55 °C for 30 s and 72 °C for 1 min [4, 30, 32]. *Trichophyton verrucosum* ATCC® 42,898™ was used as a positive control. *Staphylococcus aureus* ATCC 25923 was used as negative control and DNase and RNase-free Water were used as blank control in PCR. The PCR products were electrophoresis analyzed with 1% agarose gel, and all PCR products were sequenced.

### Phylogenetic analysis

The sequencing results were evaluated to determine the closest relatives using nucleotide BLAST. The similarity analysis was performed using the Sequence Distances method of MegAlign software. A phylogenetic analysis of sequences together with sequences of the closest relatives available in the GenBank database was conducted using the neighbor-joining method of MEGA 6.0. The sequences were submitted to the NCBI database to obtain the GenBank accession number. *Trichophyton verrucosum* ATCC 42898 was used for position controls and its GenBank accession numbers was KJ606085.1.

### Statistical analysis

All data were subjected to statistical analysis for the interpretation of the results with Graphpad prism 6 and SAS software. Risk factor analyses were performed considering locality (Yinchuan, Wuzhong, Shizuishan and Guyuan), age (calf and adult cattle), season (winter, spring, summer and autumn), and lesion sites (face, head, trunk, neck and whole body), considering a significant *p*-value < 0.05, calculated through the Chi-square test [3].

## Data Availability

The DNA sequences during the current study are available in the NCBI database. GenBank accession numbers: MT509397 (NXYC1), MT509398 (NXYC2), MT509399 (NXYC3), MT509400 (NXWZ1), MT509401 (NXWZ2), MT509402 (NXWZ3), MT509403 (NXSZS1), MT509404 (NXSZS2), MT509405 (NXSZS3), KJ816333 (NXGY1), KJ816334 (NXGY2).

## Abbreviations

SDA: Sabouraud Dextrose Agar
*T. verrucosum*: *Trichophyton verrucosum*

## References

[1] El-Tras WF, Tayel AA, Mohamed RA, El-Kordy DM, Samir A (2015). Mixed rearing correlates with the existence of Trichophyton verrucosum pathogens in humans. Dermatol Sin.

[2] Weitzman I, Summerbell RC (1995). The dermatophytes. Clin Microbiol Rev.

[3] Agnetti F, Righi C, Scoccia E, Felici A, Crotti S, Moretta L, Moretti A, Maresca C, Troiani L, Papini M (2014). Trichophyton verrucosum infection in cattle farms of Umbria (Central Italy) and transmission to humans. Mycoses..

[4] Dalis JS, Kazeem HM, Jkp K, Kwanashie CN, Yakubu B, Owolodun OA, Jambol RA (2018). Molecular characterization of dermatophytes isolated from cattle in plateau state, Nigeria. Vet Microbiol.

[5] Xiao YL, Zhang QQ (2008). Phenotype and molecular biology identification of dermatophytes. Chinese J Mycol.

[6] Yang ZF, Dong N, Yang QM, Wang Z (2009). Research progress about dermatophytosis. Modern J Anim Husbandry Vet Med.

[7] Papini R, Nardoni S, Fanelli A, Mancianti F (2010). High infection rate of trichophyton verrucosum in calves from Central Italy. Zoonoses Public Hlth.

[8] Ajello L (1962). Present day concepts of the dermatophytes. Mycopathol Mycol Appl.

[9] Grumbt M, Monod M, Staib P (2011). Genetic advances in dermatophytes. FEMS Microbiol Lett.

[10] Bassiri-Jahromi S (2013). Epidemiological trends in zoophilic and geophilic fungi in Iran. Clin Exp Dermatol.

[11] Aly R, Francisco PS (1994). California. Ecology and epidemiology of dermatophyte infections. J Am Acad Dermatol.

[12] Chermette R, Ferreiro L, Guillot J (2008). Dermatophytoses in animals. Mycopathologia..

[13] Hameed K, Riaz CF, Nawaz MA, Sms N, Grãser Y, Kupsch C (2017). Trichophyton verrucosum infection in livestock in the chitral district of Pakistan. J Infect Dev Ctries.

[14] Swai ES, Sanka PN (2012). Bovine Dermatophytosis caused by Trichophyton Verrucosum: a case report. Vet World.

[15] Dalis JS, Kazeem HM, Kwaga JKP, Kwanashie CN (2014). An outbreak of ringworm caused by Trichophyton verrucosum in a group of calves in Vom, Nigeria. Afr J Microbiol Res.

[16] Ming PX, Ti YL, Bulmer GS (2006). Outbreak of trichophyton verrucosum in China transmitted from cows to humans. Mycopathologia..

[17] Dalis JS, Kazeem HM, Kwaga JKP, Kwanashie CN (2019). Prevalence and distribution of dermatophytosis lesions on cattle in plateau state, Nigeria. Vet World.

[18] Aghamirian MR, Ghiasian SA (2011). Dermatophytes as a cause of epizoonoses in dairy cattle and humans in Iran: epidemiological and clinical aspects. Mycoses..

[19] Moretti A, Agnetti F, Mancianti F, Nardoni S, Righi C, Moretta I, Morganti G, Papini M (2013). Dermatophytosis in animals: epidemiological, clinical and zoonotic aspects. G Ital Dermatol Venereol.

[20] Courtellemont L, Chevrier S, Degeilh B, Belaz S, Gangneux JP, Robert-Gangneux F (2017). Epidemiology of Trichophyton verrucosum infection in Rennes University hospital, France: a 12-year retrospective study. Med Mycol.

[21] Wu SX, Guo NR, Liu WD (2011). Dynamic epidemiologic survey of pathogenic dermatophytes of China at 1986, 1996 and 2006. J Diagn Ther Dermato-Venereol.

[22] Mahmoudabadi AZ, Zarrin M (2008). Isolation of dermatophytes and related keratinophilic fungi from the two public parks in Ahvaz. Jundishapur J Microb.

[23] Singh I, Kushwaha RK (2010). Dermatophytes and related keratinophilic fungi in soil of parks and agricultural fields of Uttar Pradesh, India. Indian J Dermatol.

[24] De Chaumont A, Pierret C, Janvier F, Goudara Y, Kerangal X, Chapuis O (2014). Mucormycosis: a rare complication of an amputation. Ann Vasc Surg.

[25] Garmhausen D, Hagemann T, Bieber T, Dimitriou I, Fimmers R, Diepgen T, Novak N (2013). Characterization of different courses of atopic dermatitis in adolescent and adult patients. Allergy..

[26] Vennewald I, Klemm E (2010). Otomycosis: diagnosis and treatment. Clin Dermatol.

[27] Bredahl L, Gyllensvaan C (2000). Incidence and control of cattle ringworm in Scandinavia. Mycoses..

[28] Lund A, Bratberg AM, Næss B, Gudding R (2014). Control of bovine ringworm by vaccination in Norway. Vet Immunol Immuno.

[29] ElAshmawy WR, Ali ME (2016). Identification of Different Dermatophytes Isolated from Cattle, Cats and Horses Suffered from Skin Lesions. AJVS.

[30] Makimura K, Tamura Y, Mochizuki T, Hasegawa A, Tajiri Y, Hanazawa R, Uchida K, Saito H, Yamaguchi H (1999). Phylogenetic classification and species identification of dermatophyte strains based on DNA sequences of nuclear ribosomal internal transcribed spacer 1 regions. J Clin Microbiol.

[31] Moretti A, Boncio L, Pasquali P, Fioretti DP (2010). Epidemiological aspects of dermatophyte infections in horses and cattle. J Vet Med.

[32] Salari S, Ayatollahi Mousavi SA, Hadizadeh S, Izadi A (2017). Epidemiology of dermatomycoses in Kerman Province, southeast of Iran: a 10-years retrospective study (2004-2014). Microb Pathogenesis.

